# Evidence on the efficacy of small unoccupied aircraft systems (UAS) as a survey tool for North American terrestrial, vertebrate animals: a systematic map

**DOI:** 10.1186/s13750-022-00294-8

**Published:** 2023-02-13

**Authors:** Jared A. Elmore, Emma A. Schultz, Landon R. Jones, Kristine O. Evans, Sathishkumar Samiappan, Morgan B. Pfeiffer, Bradley F. Blackwell, Raymond B. Iglay

**Affiliations:** 1 Department of Wildlife, Fisheries and Aquaculture, Mississippi State University, Thompson Hall, Box 9690, Mississippi State, MS 39762 USA; 2 Geosystems Research Institute, Mississippi State University, Mississippi State, MS 39762 USA; 3 U.S. Department of Agriculture, Animal and Plant Health Inspection Service, Wildlife Services, National Wildlife Research Center, Ohio Field Station, Sandusky, OH USA; 4 Forestry and Environmental Conservation, Clemson University, Clemson, SC 29634 USA

**Keywords:** Count, Monitor, RPA, UAV, UVS, Wildlife, Remotely piloted aircraft, Unmanned aerial vehicle, Unmanned aircraft system, Uncrewed vehicle system

## Abstract

**Background:**

Small unoccupied aircraft systems (UAS) are replacing or supplementing occupied aircraft and ground-based surveys in animal monitoring due to improved sensors, efficiency, costs, and logistical benefits. Numerous UAS and sensors are available and have been used in various methods. However, justification for selection or methods used are not typically offered in published literature. Furthermore, existing reviews do not adequately cover past and current UAS applications for animal monitoring, nor their associated UAS/sensor characteristics and environmental considerations. We present a systematic map that collects and consolidates evidence pertaining to UAS monitoring of animals.

**Methods:**

We investigated the current state of knowledge on UAS applications in terrestrial animal monitoring by using an accurate, comprehensive, and repeatable systematic map approach. We searched relevant peer-reviewed and grey literature, as well as dissertations and theses, using online publication databases, Google Scholar, and by request through a professional network of collaborators and publicly available websites. We used a tiered approach to article exclusion with eligible studies being those that monitor (i.e., identify, count, estimate, etc.) terrestrial vertebrate animals. Extracted metadata concerning UAS, sensors, animals, methodology, and results were recorded in Microsoft Access. We queried and catalogued evidence in the final database to produce tables, figures, and geographic maps to accompany this full narrative review, answering our primary and secondary questions.

**Review findings:**

We found 5539 articles from our literature searches of which 216 were included with extracted metadata categories in our database and narrative review. Studies exhibited exponential growth over time but have levelled off between 2019 and 2021 and were primarily conducted in North America, Australia, and Antarctica. Each metadata category had major clusters and gaps, which are described in the narrative review.

**Conclusions:**

Our systematic map provides a useful synthesis of current applications of UAS-animal related studies and identifies major knowledge clusters (well-represented subtopics that are amenable to full synthesis by a systematic review) and gaps (unreported or underrepresented topics that warrant additional primary research) that guide future research directions and UAS applications. The literature for the use of UAS to conduct animal surveys has expanded intensely since its inception in 2006 but is still in its infancy. Since 2015, technological improvements and subsequent cost reductions facilitated widespread research, often to validate UAS technology to survey single species with application of descriptive statistics over limited spatial and temporal scales. Studies since the 2015 expansion have still generally focused on large birds or mammals in open landscapes of 4 countries, but regulations, such as maximum altitude and line-of-sight limitations, remain barriers to improved animal surveys with UAS. Critical knowledge gaps include the lack of (1) best practices for using UAS to conduct standardized surveys in general, (2) best practices to survey whole wildlife communities in delineated areas, and (3) data on factors affecting bias in counting animals from UAS images. Promising advances include the use of thermal sensors in forested environments or nocturnal surveys and the development of automated or semi-automated machine-learning algorithms to accurately detect, identify, and count animals from UAS images.

**Supplementary Information:**

The online version contains supplementary material available at 10.1186/s13750-022-00294-8.

## Background

Small unoccupied aircraft systems (UAS, also commonly referred to as uncrewed or unmanned aerial vehicle (UAV), unmanned vehicle system (UVS), unmanned aircraft (UA), remotely piloted aircraft (RPA), or drones), are powered aircraft weighing less than 24.9 kg and controlled remotely without the need for an onboard human pilot [1–3]. Data for many studies collected by occupied aircraft and ground-based methods are increasingly being replaced or supplemented by UAS as they have demonstrated promise for developing automated and standardized animal assessments [4–7]. Primary benefits of using UAS for animal studies include their ability to cover expansive areas at fine spatial and temporal resolutions [8], reduce surveyor bias and labor costs [6], access inconvenient or unsafe locations, minimize environmental impacts, including problematic animal behavior [9, 10], and increase personnel safety [11] and logistical operations compared to occupied aircraft [5, 12, 13].

To date, UAS have been used for a wide variety of studies, including animal surveys (i.e., population counts), animal behavior, movement tracking, and habitat quality assessments among diverse taxa [4], including birds (e.g., [6, 9, 10, 13–15]), mammals (e.g., [16–19]), and reptiles (e.g., [20]), in marine and terrestrial systems [12]. Many commercial UAS models and sensors are available and vary in their usefulness by survey goals and environments [12]. For example, a particular UAS model (e.g., DJI Matrice 600 Pro) might be chosen based on platform type (e.g., a multirotor over a fixed-wing), ease of use, payload capacity to carry the desired sensor, or cost [5]. Likewise, sensors vary in the type and quality of data they provide (e.g., visible vs. thermal), and these data are affected by survey conditions and sensor capabilities. Such ‘off-the-shelf’ UAS packages further support widespread use of UAS and exploration of new applications [21]. However, the recent rapid increases in UAS use, improvements to associated model, sensor, and computer vision technologies, and a common interest to incorporate UAS in a myriad of situations have a relatively sparse foundation of scientific investigations. UAS studies tend to build toward common approaches, but few studies share complete methods or operating guidelines to effectively incorporate this new technology for future use. For example, justifications for selecting UAS models and sensors are typically not offered in studies, and standardized reporting mechanisms have only recently emerged [22], but might not always be followed. Further confounding the use of UAS for counting animals is a lack of bias-corrected estimates (i.e., estimates accounting for sampling errors such as false positives or negatives), despite calls for such studies [23]. Despite the myriad of studies highlighting various opportunities and limitations of UAS applications for monitoring animals, information leading to best practices for such applications, including accurate estimates (e.g., bias-corrected estimates), have yet to be amalgamated into a single literature resource [23, 24]. To better use available information, we began the development of a systematic map [25].

A systematic map approach is a repeatable process that can answer broader questions than systematic reviews by collating, describing, and cataloging evidence related to the topic of interest [26]. A preliminary literature search returned no existing systematic maps pertaining to monitoring animals with UAS. A systematic map is therefore critical in setting the necessary foundation for improved scientific rigor informing UAS applications in animal monitoring. Most published reviews focus on future UAS use [12], overall accomplishments and challenges [6, 7], animal behavioral responses [27], or general research summaries [5, 14]. A systematic map, however, permits evaluation and summarization of past and current UAS applications for animal monitoring among UAS model and sensor technologies, taxonomic and geographic scopes, flight conditions and operational considerations, spatial distributions of UAS applications, and reported technical benefits and pitfalls. From this generated body of evidence, standard reporting mechanisms, selection criteria, and applications emerge to complement recent efforts (e.g. [22],) and describe potential bias associated with UAS animal surveys.

Our objective was to develop a systematic map that consolidates evidence in the aforementioned areas affecting and pertaining to the use of UAS to monitor animals in terrestrial environments worldwide. Considering the rapid expansion of UAS technology, this systematic map will inform future researchers, practitioners, and other end users planning to apply UAS in terrestrial ecosystems to monitor animals, of the best practices based on the current state of knowledge. Whereas much literature has been published in marine systems [e.g., 28, 29], we focused our systematic map on vertebrates in terrestrial systems only. Although our systematic map was not limited in geographic scope, our species-specific search terms were limited primarily to vertebrate taxa of North America, due to interest of collaborators, stakeholders, and funders whose goal involves understanding the role of UAS in wildlife monitoring applications to aid in understanding and mitigating animal-vehicle collisions on and around airports [30, 31]. This systematic map elucidates major knowledge clusters (well-represented subtopics that are amenable to full synthesis by a systematic review) and gaps (unreported or underrepresented topics that warrant additional primary research) among UAS-animal related studies, as well as summarizes current applications in a repeatable framework for future UAS assessments.

## Stakeholder engagement

The systematic map protocol [25] and subsequent map were developed in collaboration with Mississippi State University Department of Wildlife, Fisheries and Aquaculture (MSU-WFA), the United States Department of Agriculture (USDA) Animal and Plant Health Inspection Service, Wildlife Services, including its Airport Wildlife Hazards Program, UAS Working Group, and National Wildlife Research Center (NWRC), and the Federal Aviation Administration (FAA). All entities listed above were considered stakeholders. Authors were members of these entities except for FAA, whose senior members reviewed the systematic map protocol [25] and the final systematic map. The main authors discussed and refined the scope and objective of the systematic map during initial project planning meetings. The findings will be of direct interest to each stakeholder in considering methods by which UAS might compliment current survey practices on and near airports [32], as well as providing new insights to similar applications by other stakeholders in academia, research, industry, and other government agencies worldwide.

## Objective of the review

The objective of this review was to determine the current state of knowledge regarding how UAS have been used to monitor terrestrial, vertebrate animals. While UAS have been used to collect images for automated and standardized animal assessments, the extent to which they have been used has not been fully summarized, including study specifics such as efficacy and accuracy. This review provides a quantitative, repeatable process to develop objective methods for monitoring and counting animals in terrestrial environments. Our specific goal was to provide a comprehensive, catalogued state of knowledge surrounding UAS models and sensors used to monitor terrestrial animals.

### Primary question

What evidence exists on the efficacy of UAS as a survey tool for terrestrial, vertebrate animals?

### Secondary questions

1. What UAS models and sensors are used most for monitoring terrestrial, vertebrate domestic and wild animals (hereafter, “animals”)?
2. What are the common statistical approaches and field methodologies of UAS applications for monitoring animals?
3. What are the common geographic ranges, vegetation types (i.e., land covers), species or species groups of UAS applications for monitoring animals?
4. What factors affect or are perceived to affect accuracy (i.e., sampling bias) in counting animals in UAS imagery (e.g., animal size, behavior, land/water cover, weather, or light conditions, etc.)?
5. What are the common constraints of UAS for monitoring animals (e.g., UAS models and sensors, government restrictions, sensor calibration, expense, battery life, etc.)?

### Components of the primary question

Population (P): All terrestrial vertebrate wildlife species and domestic animals (inclusive of humans, animals that commonly occur in aquatic systems such as shorebirds, waterfowl, turtles, or crocodilians, or animals that commonly occur in aerial systems such as birds or bats).

Index test (I): The technology of interest (UAS).

Target condition (T): Presence or abundance of population (i.e., study goals, but not type or quality of data obtained).

## Methods

### Deviations from the protocol

**Fig. 1:**
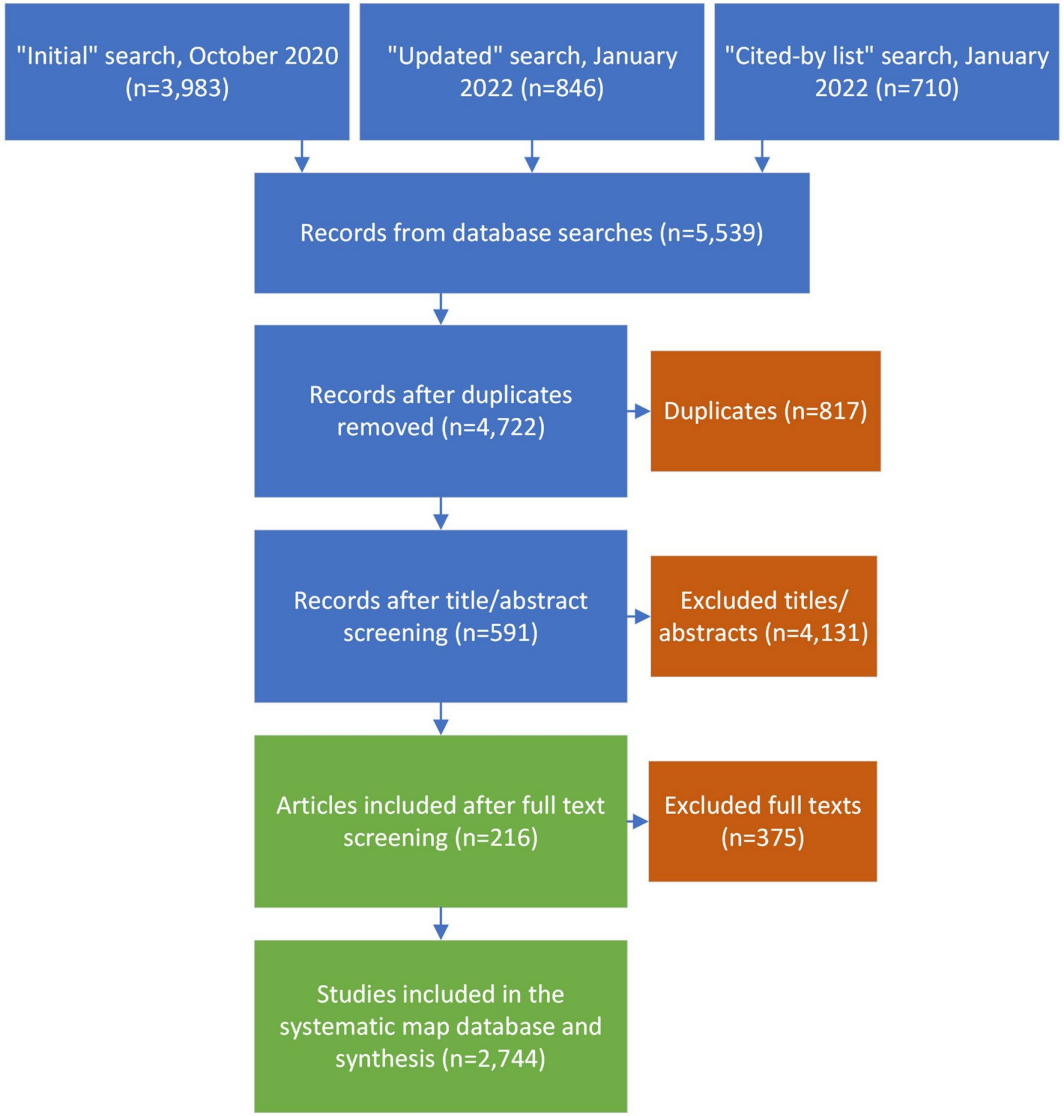
Schematic of mapping stages, including search and screening stages, leading to final articles and studies included in this systematic map of using UAS to survey animals

We only searched through the “cited-by lists” of the top 10, most-cited articles instead of the top 20, because the top 10 articles yielded > 700 additional articles added to the literature search prior to duplicate removal (Fig. 1; Additional file 5). We reworded several secondary questions, and rephrased secondary question 3 to make it more general and applicable for a systematic map. We removed secondary question 6 (“What are the suggested statistical approaches and characteristics of sampling designs that can lead to a consistent set of best practices to avoid, reduce, and correct sampling bias from UAS aerial imagery while still achieving project goals?”) as it was deemed too specific for a systematic map, and more appropriate for a systematic review.

### Searching for articles

#### Search terms and languages

All searches were performed in English (i.e., search terms were only in English), and only studies published in or translated to English were included due to the languages understood by the systematic map review team. Some search terms were specific to North America due to stakeholder-driven requirements, but the geographic scope of studies returned or included in this systematic map was not limited.

When applicable, search terms were truncated and a wildcard (*) added at the end of the root word to include all alternate forms of root words to account for alternative spelling or hyphenation (e.g., estimat* to account for estimate, estimation, estimating, estimates, estimated). Dollar signs ($) were added when applicable to designate the addition of one extra character. Quotation marks were placed around some multiple word terms to allow for the search of exact phrases. All search terms and phrases were combined using the Boolean operators AND or OR. All searches were conducted on the article title, abstract, and keywords except for Google Scholar, which only allows searches at title or full text.

#### Search string

We conducted a test search on October 20th, 2020, in multiple databases, as part of a scoping exercise to help build search terms and ensure the correct use of operators to yield the best performance in returning results. The following search string produced efficient results, with > 99.99% accuracy among two searchers (1502 in Web of Science, 2041 in Scopus, 172 in Wildlife and Ecology Studies Worldwide, and 144 in Proquest Dissertations and Theses):

[(*UAS$ OR UAV$ OR UVS$ OR RPA$ OR "unmanned aerial system$" OR "unmanned aerial vehicle$" OR "unmanned vehicle system$" OR "unmanned aircraft" OR "remotely piloted aircraft" OR "unoccupied aerial vehicle$" OR "unoccupied aerial system$" OR “drone$”*) **AND** (animal* OR avian OR bird* OR mammal* OR reptil* OR wildlife OR carnivor* OR cattle OR deer OR furbearer* OR livestock OR mesocarnivore* OR shorebird* OR ungulate* OR waterbird* OR waterfowl* OR alligator* OR blackbird* OR cow* OR coyote* OR dove* OR eagle* OR geese OR goose OR gull* OR hawk* OR hog* OR owl* OR passeri* OR pig* OR quail* OR raptor* OR snake* OR starling* OR "terrestrial vertebrate$" OR turkey* OR turtle* OR vulture*) **AND** (abundance* OR assess* OR count* OR estimat* OR monitor* OR population*)].

#### Publication databases

Relative to online publication databases, we used the prior search string to perform searches through Mississippi State University Libraries on the Web of Science search platform, Scopus, Wildlife and Ecology Studies Worldwide, and Proquest Dissertations and Theses. Databases included in the Web of Science search platform are the Core Collection, SciELO Citation Index, and Zoological Record. Databases were chosen based on the comprehensive coverage of published literature.

#### Internet searches

An internet search was conducted with the same search string as above using Google Scholar on article titles, and the first 100 results, sorted by relevance, were added to the list of search records.

#### Supplementary searches

A search for non-peer reviewed literature was conducted by request through a professional network of collaborators (e.g., USDA, FAA, United States Department of Defense, The Wildlife Society) and publicly available websites including Research Gate (http://www.researchgate.net), and Twitter (http://twitter.com), as well as the Ecological Society of America mailing list (https://www.esa.org/membership/ecolog/; Additional file 2). No additional articles were added to our database/internet searches from this request. We also scanned reference lists of all articles included after full-text screening for our search terms, review articles, and “cited by lists” for the top-10 most cited articles, to search for relevant, but missed articles which were then added to the search results. Government reports, white papers, grey literature, and information from conference proceedings not returned from our primary searches were included and underwent all screening.

#### Search settings

To help control for bias injected into searches by learning algorithms of internet browsers, browser history and cookies were disabled when conducting all searches. The search team used “InPrivate” or “incognito” mode and did not access any electronic accounts. All searches were conducted by one search team member because consistency was checked during the scoping stage.

#### Comprehensiveness of the search

To evaluate the comprehensiveness of the search strategy, we compiled a list of 41 benchmark articles (Additional file 3) from and including two recent reviews. We included articles listed under the “wildlife research and management” section of one review [35] and under the “UAS” section of another review [36]. Articles deemed outside the realm of our search (e.g., marine, non-UAS, etc.) were excluded from our list of benchmark articles. All articles from the list of benchmark articles were found in our search justifying no amendments to our search string.

### Article screening and study eligibility criteria

#### Screening process

We screened article records using a hierarchical approach in the order of title, abstract, and full text. Information for all article records returned from our search was first imported to Rayyan QCRI to resolve duplicates (i.e., identical articles returned from multiple search databases) and screen for relevance (i.e., those using UAS to monitor terrestrial animals) [35]. Any article records where a decision could not be made to either include or exclude were passed to the next stage of screening (i.e., records not determined as relevant at the title stage moved forward to the abstract stage of screening). After duplicate removal one reviewer, JAE, screened all articles at title and abstract level (n = 4722). An additional reviewer, MFC, screened a subset of 847 articles at title and abstract level, and a Cohen’s kappa coefficient of 0.95 indicated consistency (Additional file 9). Exclusion decisions for articles at the title and abstract level are included under the “notes” column of Additional file 5. All articles passing to the full-text screening stage were exported from Rayyan QCRI and saved as a.csv file. Article records that moved to the full-text stage were accessed and downloaded as PDF files using licenses through Mississippi State University, Google Scholar, USDA, or personal inquiries with corresponding authors of articles. The pdf files were uploaded to a shared Mendeley group folder for full-text screening by JAE, EAS, and LRJ [36]. Reviewers did not screen nor extract data from any works they had authored. We used tools in Mendeley to record exclusion reasonings and highlight relevant data for included articles. A subset of 10% of article records (n = 39) from the initial search (384 articles passed to full text stage of review) were independently assessed by all reviewers at the full-text stage to determine reviewer agreement and a Cohen’s kappa coefficient of ≥ 0.90 indicated consistency (Additional file 9) [37]. Then, JAE screened 247 articles, EAS 171 articles, and LRJ 134 articles. A total of 216 articles met our inclusion criteria at full text for data extraction (Fig. 1; Additional files 6, 7, & 8). Exclusion decisions for articles at the full text level are included under the “remarks” field in the “tblMaster_Table” table of the resultant database (Additional file 6 and 7).

### Eligibility criteria

*Eligible population*: All terrestrial vertebrate animals including, wildlife, and domestic animals were deemed as eligible subjects.

*Eligible index test*: All studies using UAS technology to monitor (i.e., identify, count, estimate, etc.) eligible populations were included.

*Eligible target condition*: All studies reporting presence or abundance of animal population were included. Studies observing strictly behavior or deterrence of animals in response to UAS, or opinion, comment, review, or discussion type manuscripts were excluded from the systematic map.

*Eligible study designs*: All studies designed to count or monitor terrestrial vertebrate animals were deemed as eligible studies. We only evaluated studies published in or translated to English. We did not apply any date restrictions (i.e., all articles from the beginning of database history through the date of the search).

### Study validity assessment

No formal study validity assessment was conducted, as this study was not intended to assess study quality but provide consolidated information necessary to gain a broad perspective of existing research about UAS used to monitor animals.

### Data coding strategy

Data extracted from studies included a variety of aspects from categories, including bibliographic information, study characteristics, index information, population information, and target information (Additional file 4). Data were recorded in a Microsoft Access relational database to reduce data redundancies following a standard operating procedure (Additional file 10). Reviewers independently extracted data from a subset of 17 articles included from the initial search (17 of the 39 articles assessed at full text stage for inclusion) to determine accuracy of extracted information, and a Cohen’s kappa coefficient of ≥ 0.60 to indicated consistency [37]. Reviewers discussed the extracted information to ensure accuracy moving forwards and if, at any time, one reviewer encountered data that was questionable, all reviewers met to discuss a collective decision. Then JAE extracted data from 78 articles, EAS 95 articles, and LRJ 26 articles. Potential data duplicates were noted and screened to ensure duplicated data were not entered into the database. For example, theses/dissertations were checked against published peer-reviewed articles to ensure that data were not entered twice (e.g., [21, 38]); in this case the peer-reviewed article was the record for included data. In cases of two peer-reviewed articles with duplicated data, the peer-reviewed article that was published first was the record included for data extraction (e.g., [39, 40]).

### Study mapping and presentation

In this systematic map, we describe the process and include a summarized narrative and numbers of articles or studies for each stage of the inclusion process and extracted data. We define articles as the published format of research, and studies as the unique investigation within articles [26]. Some articles may have multiple studies extracted for each metadata field (e.g., an author flew multiple different UAS models), thus, there may be more studies for each metadata field than the number of articles included in the systematic map. Because some articles failed to report data, we record these as unknown for each metadata field within our database. The percentages reported within data categories were calculated from the total number of studies with known data including unknown studies for each metadata field separately. The final database with all extracted data, was produced as a Microsoft Access database (Additional file 6), a Microsoft Runtime database (Additional file 7), and a Microsoft Excel spreadsheet (Additional file 8). All three contain the same information but in different formats. Users without Microsoft Access on their computer can download the free version of Microsoft Access runtime from https://www.microsoft.com/en-ca/download/details.aspx?id=50040. Queries from the final database were used to produce tables, figures, geographic maps, and word clouds, which accompany the narrative review, answering our primary and secondary questions. In the case that data was entered in open text fields, we checked each field to ensure consistent terminology (Additional file 6 & 12), and then used Program R [41] to reduce text and generate word clouds. Subtopics and questions identified through the course of the systematic mapping process are described in detail. The tables, figures, geographic maps, and word clouds of study frequencies were used to identify major knowledge clusters and gaps, and implications for informing policy/management and future research are discussed.

## Review findings

### Review descriptive statistics

#### Searching and screening

Search efforts for articles were conducted in three waves: an initial search in October 2020, an updated search in January 2022, and a search of “cited-by lists” in January 2022 (Fig. 1; Additional file 5). After duplicate removal, title and abstract screening was performed on 4722 articles, and full text screening was performed on 591 articles (Fig. 1; Additional file 6). A total of 216 articles met our inclusion criteria for data extraction after full text screening (Fig. 1; Additional file 6, Additional file 7, Additional file 8). The total number of studies (i.e., unique investigations [26]) from data extraction was 2744.

### Mapping the quantity of studies relevant to the primary and secondary questions

We completed our primary objective by creating and populating a database of all available evidence for our primary question “What evidence exists on the efficacy of UAS as a survey tool for terrestrial, vertebrate animals?”. In this database, users can easily query, filter, or search for the population, index test, or target condition (PIT) elements desired. We have provided this database in three file formats: (1) a Microsoft Access database (Additional file 6), (2) a Microsoft Access Runtime database (Additional file 7), and (3) a Microsoft Excel spreadsheet (Additional file 8). The Microsoft Access versions are easier to search for studies by using predefined and newly created queries and allows for users to see the complex relationships among different data tables.

We completed our secondary objective by populating additional fields from our data coding strategy in the database to provide evidence to answer our 5 secondary questions. Evidence for each of these questions can be found by running pre-defined queries in the database. This approach helped to further describe the evidence existing on the efficacy of UAS as a survey tool for terrestrial vertebrates by defining major knowledge clusters and gaps.

### Description of knowledge clusters and gaps in studies

#### Publication type and chronological distribution of studies

Included articles represented six publication types, of which most were published in peer-reviewed journals (n = 176, ~ 81%). Remaining articles were conference proceedings (n = 19, ~ 9%), theses/dissertations (n = 13, ~ 6%), official reports (n = 5, ~ 2%), book chapters (n = 1, < 1%), and magazine/news article (n = 1, < 1%).

**Fig. 2:**
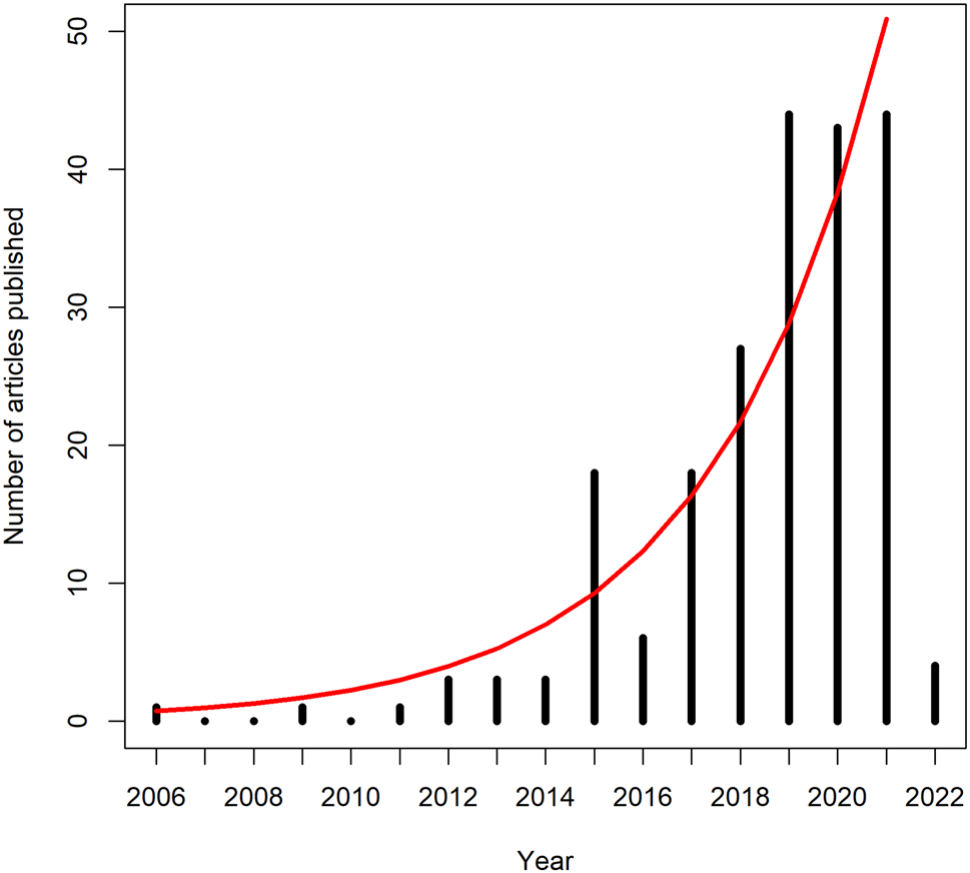
Number of articles published each year for monitoring animals using UAS. The bar for 2022 is much lower due to the search being conducted on January 3rd. The red line denotes an exponential growth curve based on the publication data

The number of articles per year generally increased exponentially over time, a trend that differs from the total number of peer-reviewed publications per year [42]. Articles leveled off from 2019–2021 between 40 and 45 publications (Fig. 2). The earliest included article was published in 2006, and there was a large uptick in articles in 2015 and 2017. Because the updated and cited by searches were conducted on January 3^rd^, 2022, there were 4 articles published with 2022 dates, hence the incomplete bar for 2022. However, we expect this trend of increasing articles will continue into the future like other emerging technologies for animal monitoring, such as camera trapping (e.g., [43]).

#### What UAS models and sensors are used most for monitoring animals?

**Fig. 3:**
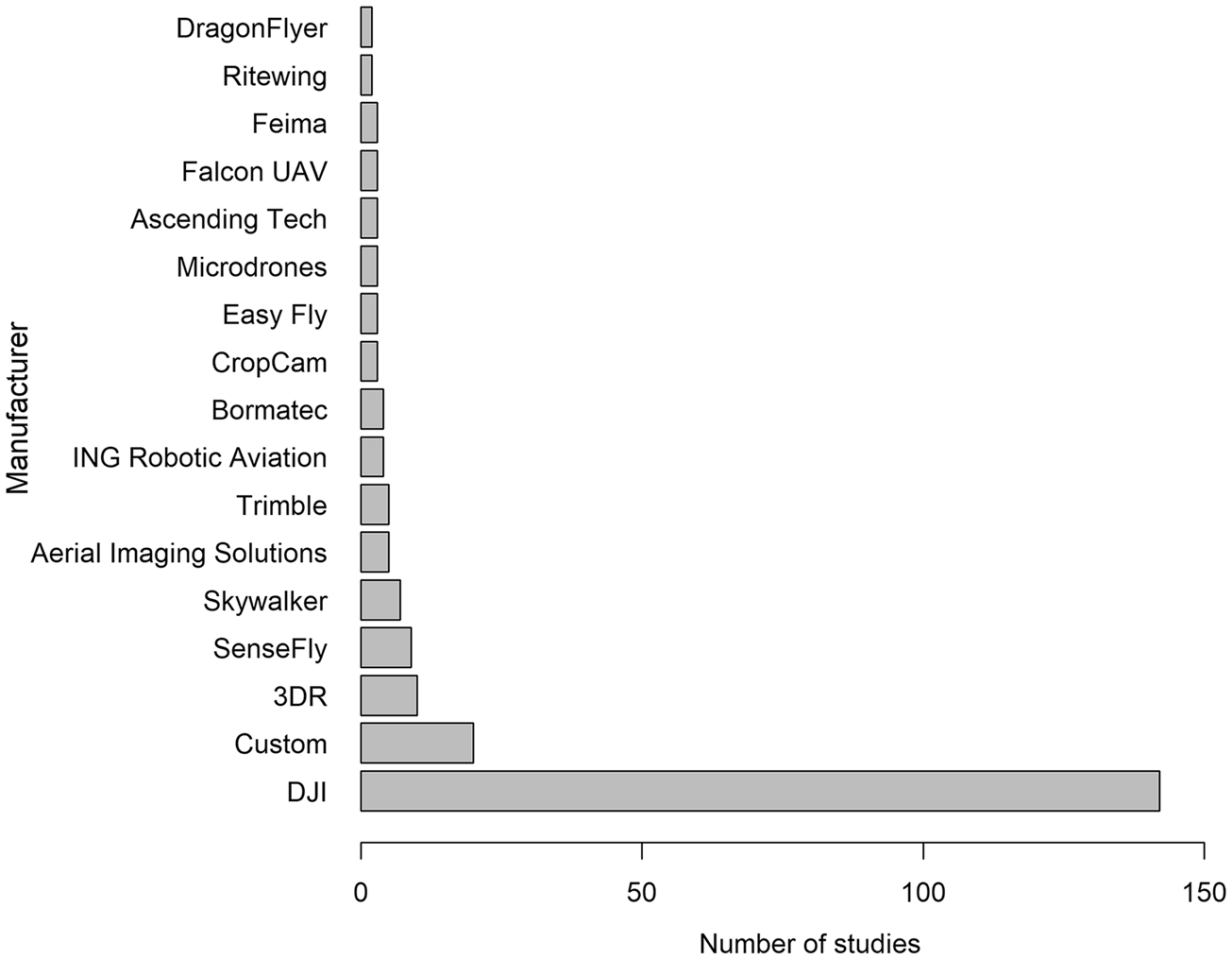
Number of studies using UAS to survey animals by each UAS manufacturer. Only manufacturers whose equipment was used in > 1 study are shown here

**Fig. 4:**
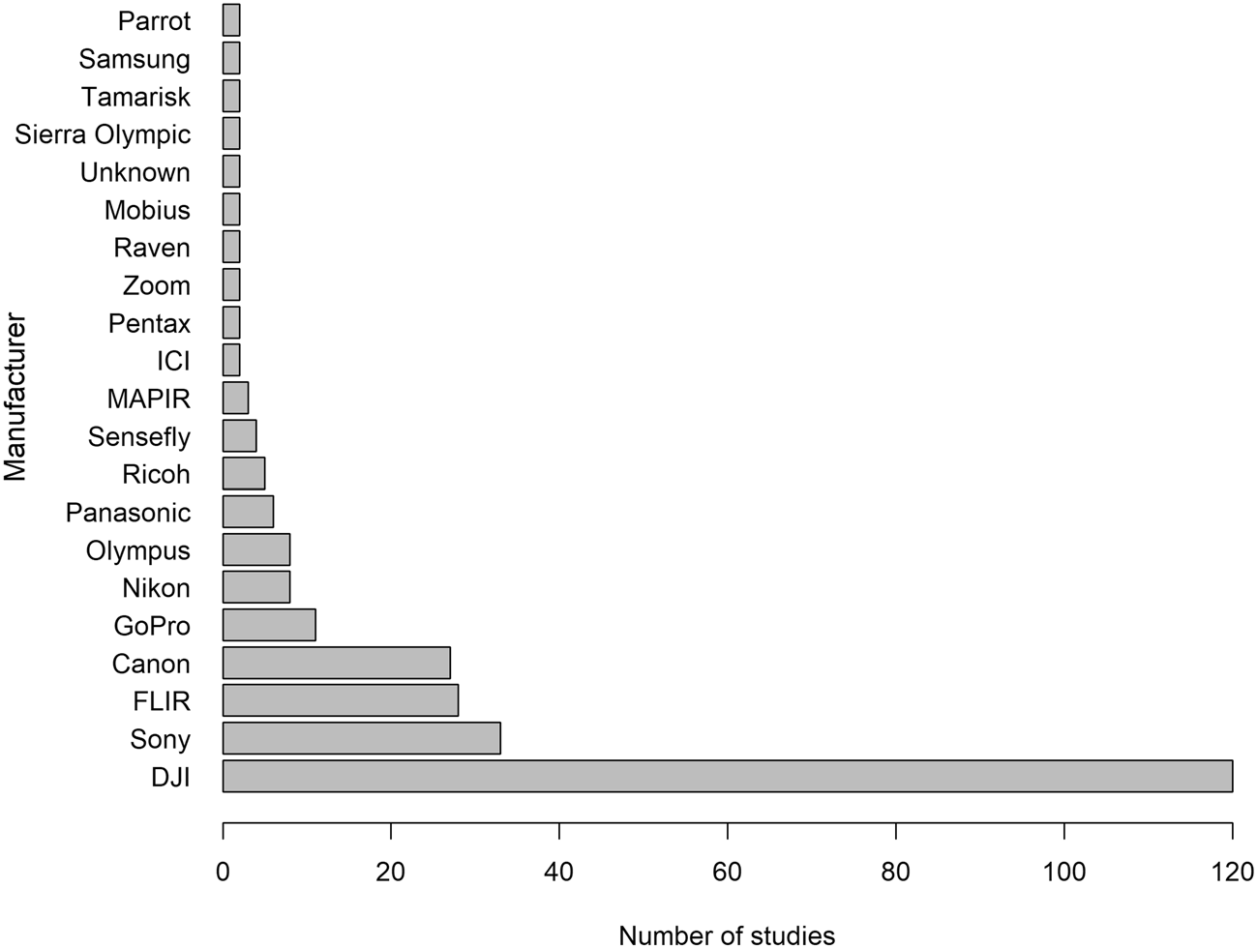
Number of studies using UAS to survey animals by each sensor manufacturer. Only manufacturers whose equipment was used in > 1 study are shown here

We recorded a total of 109 different UAS models from 50 manufacturers (Fig. 3) in 262 studies and 126 different sensors from 34 manufacturers (Fig. 4) in 262 studies. Some studies used custom-built UAS models (n = 20, 8%; e.g., [44, 45]), but no sensors were custom-built. Only one study did not report the UAS model, and one additional study did not report UAS manufacturer nor model. Likewise, three studies did not report the sensor model, and two additional studies did not report the sensor manufacturer nor model. The majority of UAS models (n = 142, 54%) and sensors (n = 120, 46%) were manufactured by DJI (Figs. 3, 4; SZ DJI Technology Co., Ltd., Shenzen, China). Imaging sensors were used most (n = 259, 99%), but three studies (1%) used audio/sound recording sensors (e.g., [46, 47]). Most imaging sensors were RGB color capturing cameras (n = 191; 73%), but 49 were thermal (19%) and 19 were both RGB and thermal (7%).

#### What are the common statistical approaches and field methodologies of UAS applications for monitoring animals?

Various methodologies were used in UAS flights among studies including how the UAS was controlled, flight software used, altitude above ground level (AGL) during flights, and flight pattern. For control type, autonomous flights occurred in most studies (n = 118, 52%), followed by manual flights (n = 45, 20%), both autonomous and manual (n = 4, 2%), and an additional 58 studies (26%) did not report how the UAS was controlled. There were 31 different types of software used, but most articles (n = 114; 51%) did not report their flight software, and one study reported no software was available for their UAS model at the time. Among studies, 87 different AGLs were reported, ranging from 3–800 m AGL, with 85 articles flying at multiple AGLs, and 16 studies (4%) not reporting the AGL for flights. Most (n = 300 studies, 80%) flew < 122 m AGL (400ft), the maximum allowable AGL under current FAA part 107 regulations for the United States [2], but 58 studies (16%) flew > 122 m AGL. There were seven different reported flight patterns, and 59 studies (25%) did not report flight pattern. Of studies that reported a flight pattern, lawnmower (i.e., parallel transects) was predominant (n = 92, 39%), followed by transect (n = 40, 17%), point (n = 25, 11%), freestyle (typically manual flights; n = 10, 4%), grid (n = 8, 3%), zigzag (n = 1, < 1%), and rosette (n = 1, < 1%).

Various methodologies associated with the sensors and data processing were used among studies, including sensor calibration in the field before use, preprocessing before analysis (e.g., imagery transformed into orthomosaics, photograph corrections, etc.), and data analysis. Most studies did not report whether field calibration was conducted (n = 195; 89%). Of the 24 studies that reported presence/absence of field calibration, 14 studies (6%) conducted field calibration and 10 studies (5%) did not. Most studies performed some type of data preprocessing such as creating image mosaics (n = 128, 59%), 49 studies (22%) performed no data preprocessing, and 42 studies (19%) did not report whether preprocessing was conducted or not. Most studies utilized humans to analyze images (n = 141, 65%), but 35 used solely automated computer classification (16%), and 30 used both humans and computers (14%). Twelve studies (5%) did not report how images were analyzed.

Information reported regarding statistical analyses included the type of analysis used, comparison of aerial data to other methods, and whether the raw data were available. Most studies used descriptive statistics with no formal analyses (n = 146; 61%; Additional file 11). Most studies did not compare UAS data to any other data (n = 94; 42%), but those that did compared them to data collected on the ground (n = 86, 38%), to another UAS (n = 36, 16%), to an occupied aircraft (helicopter [n = 5, 2%]; plane [n = 3, 1%]), or to satellite imagery (n = 1, < 1%). Only seven of the 216 articles (3%) provided links to data repositories.

Data on individual study methods including flight time of day and whether ground control points and ground truthing were used were also available. Most studies conducted flights during the day (n = 193, 87%), but 25 studies conducted flights at night (11%), and one study during both day and night (< 1%). Four studies did not report when flights were conducted (2%). Most studies did not report whether ground control points were used in their studies (n = 145; 66%), but 54 studies reported not using ground control points (25%) and 20 studies did use ground control points (9%). Likewise, most studies did not report whether their UAS survey efforts had ground truthing conducted (n = 111; 51%). Of those that did report ground truthing, 63 studies did not ground truth (29%) and 45 studies did ground truth (20%).

#### What are the common geographic ranges, vegetation types (i.e., land covers), species or species groups of UAS applications for monitoring animals?

**Fig. 5:**
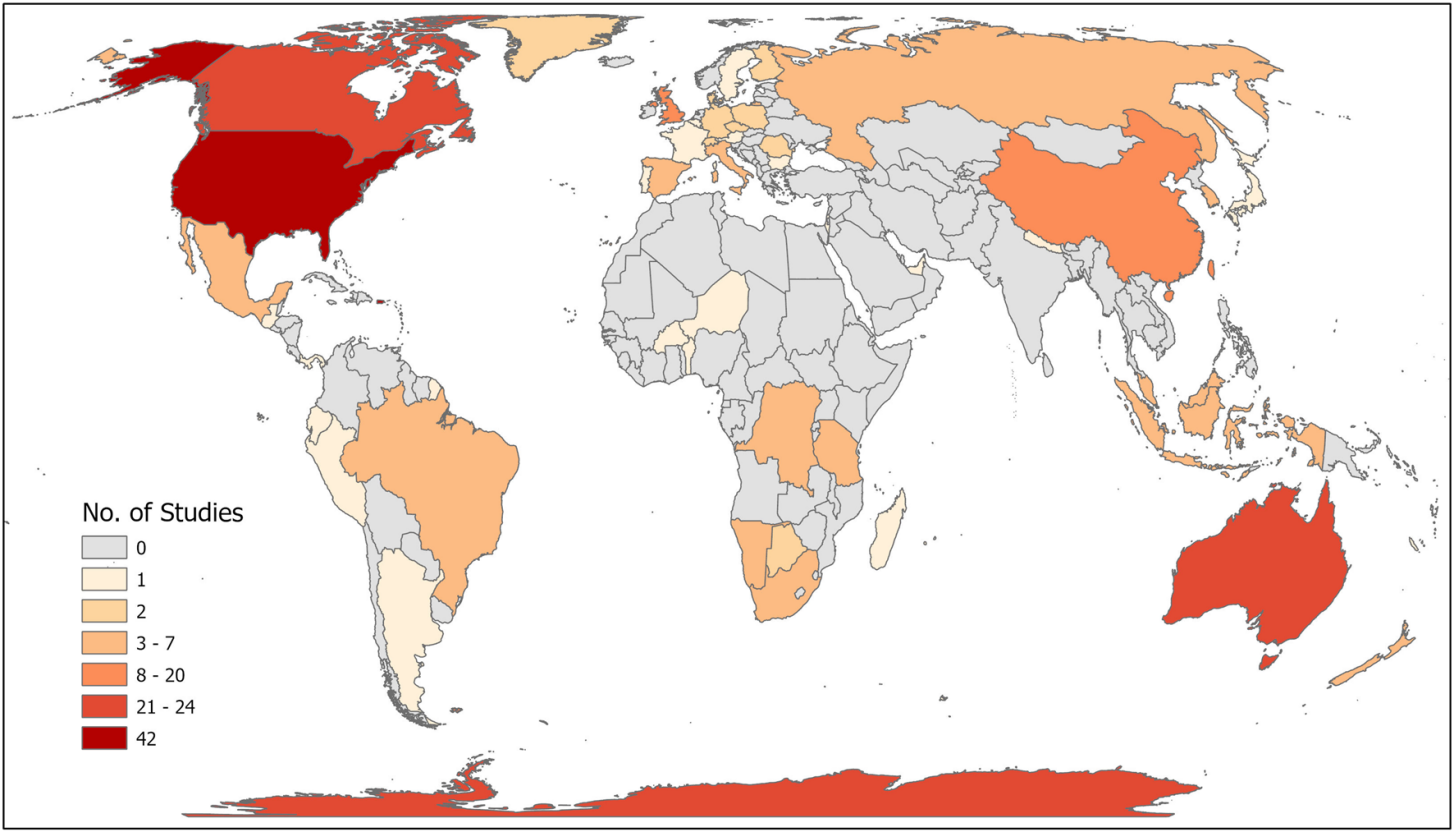
Distribution of UAS animal survey studies by country in which the study took place

There were 219 studies from 46 different countries (Fig. 5; some articles included data from multiple countries), and three additional studies (1%) did not list where the study took place. Study representation was uneven, with four countries accounting for nearly half of the studies (n = 109, 49%; Fig. 5) including the United States of America (n = 42, 19%), Canada (n = 24, 11%), Australia (n = 22, 10%), and Antarctica (n = 21, 9%; independent continent not governed by any country under Antarctic Treaty of 1959 [48]; Fig. 5). Studies were conducted in 16 different land cover types, and five studies did not report information about land cover. The top land cover types were grass (n = 52, 22%), wetland (n = 41, 18%), forest (n = 41, 18%), beach (n = 19, 8%), bare (n = 19, 8%), water (n = 16, 7%), and rock (n = 14, 6%).

**Fig. 6:**
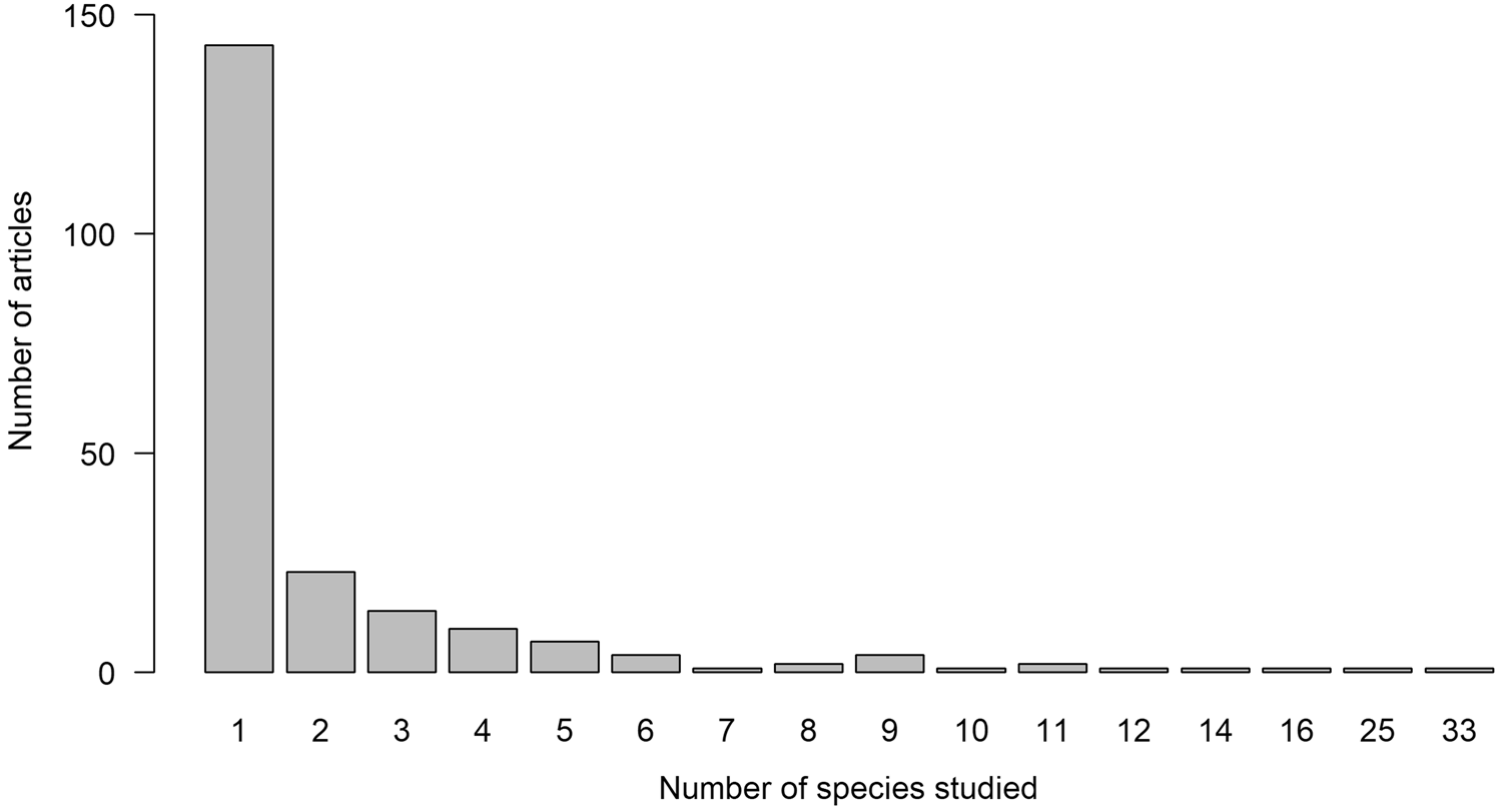
Number of published articles and number of species studied in each article for studies monitoring animals with UAS. Most articles only studied 1 species

Taxonomic information was presented at the species level in most studies (n = 285, 94%), but some studies only identified taxa to genus (n = 10; 3%), family (n = 8; 3%), or order (n = 1; < 1%). A total of 285 species, 81 families, and 4 classes were studied among articles. Animal classes were represented by 187 birds in 98 studies, 103 mammals in 113 studies, 13 reptiles in 11 studies, and 1 amphibian in 1 study. The number of species/genera (some studies could not identify to species but could identify to genus) studied in each article ranged from 1–33, but most studies were conducted on only 1 species or genera (n = 143; 66%; Fig. 6). The top 15 most studied species (n > 5 studies) consisted of 6 species of mammals and 9 species of birds (Table 1).

**Table 1:** Taxonomic information and total number of studies for the 15 most studied species monitored with UAS

Class	Family	Scientific name	Common name	# Studies
Birds	Spheniscidae	*Pygoscelis antarcticus*	Chinstrap Penguin	10
Mammals	Cervidae	*Odocoileus virginianus*	White-tailed Deer	9
Mammals	Phascolarctidae	*Phascolarctos cinereus*	Koala	8
Mammals	Bovidae	*Bos taurus*	Domestic Cattle	8
Birds	Anatidae	*Anas platyrhynchos*	Mallard	8
Birds	Ardeidae	*Ardea alba*	Great White Egret	7
Birds	Spheniscidae	*Pygoscelis adeliae*	Adelie Penguin	7
Mammals	Phocidae	*Halichoerus grypus*	Grey Seal	7
Mammals	Phocidae	*Mirounga leonina*	Southern Elephant Seal	6
Birds	Anatidae	*Anas acuta*	Northern Pintail	5
Birds	Anatidae	*Anas crecca*	Common Teal	5
Birds	Ardeidae	*Ardea cinerea*	Grey Heron	5
Birds	Spheniscidae	*Pygoscelis papua*	Gentoo Penguin	5
Birds	Phalacrocoracidae	*Leucocarbo atriceps*	Imperial Shag	5
Mammals	Hippopotamidae	*Hippopotamus amphibius*	Hippopotamus	5

#### What factors affect or are perceived to affect accuracy (i.e., sampling bias) in counting animals in UAS imagery?

**Fig. 7:**
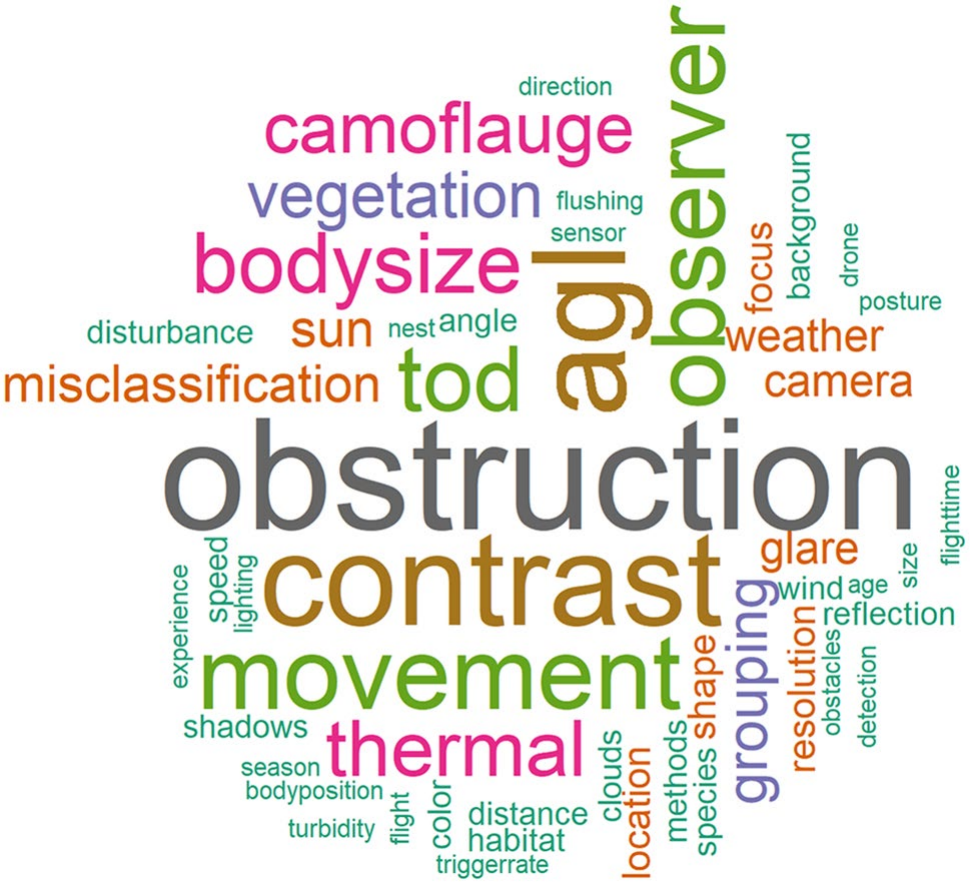
Word cloud for the most frequently used words to describe the factors affecting bias in studies using UAS to survey animals

A total of 50 primary factors were listed in 96 studies that either did or could bias detection or accuracy in counting animals via UAS (Fig. 7, Additional file 12). Additionally, most studies did not list any factors contributing to bias (n = 130; 58%). The most frequent words used to describe factors affecting bias (n ≥ 10 occurrences) were “obstruction” (n = 21), “AGL” (n = 17), “contrast” (n = 17), “observer” (n = 13), “movement” (n = 12), “time of day (TOD)” (n = 11), and “body size” (n = 11; see Additional file 12 for frequency and definition of all words).

#### What are the common constraints of UAS for monitoring animals?

**Fig. 8:**
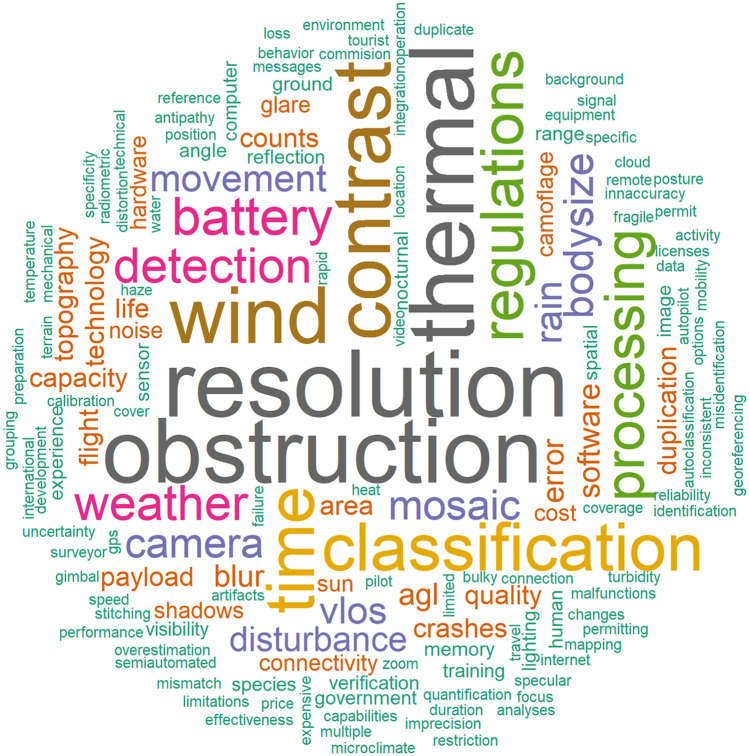
Word cloud for the most frequently used words to describe constraints in studies using UAS to survey animals

A total of 152 primary terms were listed as constraints in 126 articles (Fig. 8, Additional file 12) and an additional 90 articles listed no constraints (42%). The most frequent words used when describing constraints (n ≥ 10 occurrences) were “resolution” (n = 22), “obstruction” (n = 21), “thermal” (n = 21), “contrast” (n = 18), “wind” (n = 18), “time” (n = 15), “classification” (n = 15), “regulations” (n = 13), “processing” (n = 12), “battery” (n = 11), “detection” (n = 10), and “weather” (n = 10; see Additional file 12 for frequency and definition of all words).

### Limitations of the map

One limitation of this map is that many of the search terms regarding the population of interest were specific to North America (due to stakeholder interest). For example, our search terms included “vulture”, “hawk”, and “raptor” but not “buzzard”. Additionally, only articles published or translated to the English language were included. There may be articles with search terms outside of our area of interest that may not have been returned in our search, or articles published in other languages that were not included due to the need for translation. Hence, articles published in journals in non-English speaking countries are more likely to have not been returned in our search. However, there were 37 articles at the title/abstract level and 48 articles at the full-text level that were returned from our search but were not included because they were not translated to English. Another limitation was that most search terms were limited to general terms for animals and just a few species common names. There was no feasible way to include all common and scientific names for > 30,000 terrestrial vertebrates [49], or even just those in North America, as search terms. Thus, our search potentially missed some species-specific articles. Further, the updated search was conducted in January 2022. Any literature published between then and the publication of this article are not included here.

Some studies evaluated the behavioral response of animals to UAS or used UAS to record animal behavior [9, 10]. These were not within the scope of our target condition and were not included in this systematic map. But these studies still relate to animal monitoring with UAS and potentially warrant a future systematic map or review.

We also acknowledge that the use of UAS to monitor animals is rapidly evolving and increasing, as shown by the number of articles published over time (Fig. 2). Many new terms may become more common in the literature and terminology is subject to change, such as the FAA drone advisory committee’s recommendation for changing the terminology for UAS from “unmanned” to “uncrewed”, which occurred between our first and second literature search [3, 50]. Research efforts are ever-expanding in the diversity of taxa monitored and new technology applications such as computer vision for reviewing imagery [51]. While few, if any, systematic map or review efforts can capture and summarize such growth in a timely manner, it is another limitation of this map but also justification for revisiting this topic sooner than later.

## Conclusions

Our systematic map provides a comprehensive database consolidating the evidence on the efficacy of UAS as a survey tool for terrestrial, vertebrate animals. Our database, consisting of dozens of metadata fields, is an efficient tool for finding and consolidating this literature. Most of these metadata fields were unevenly distributed as shown in the above descriptions of knowledge clusters and gaps answering our secondary questions. From this systematic map and associated database, information can be easily found to address many knowledge clusters suitable for full systematic review, knowledge gaps warranting future research efforts, and potential policy or management decisions.

### Implications to inform policy/management

Our results indicate that the literature for the use of UAS to conduct animal surveys has expanded immensely in a short period of time but is still in its infancy. Early studies prior to 2015 were typically represented by a few pioneers using expensive, fixed-wing, custom-built or assembled platforms that were not commercially available and relied on low-resolution, non-stabilized (i.e., non-gimballed) sensors [7, 21, 52, 53]. Since 2015, technological improvements to commercially available platforms, particularly quadcopters, high-resolution sensors, and software became widespread and relatively inexpensive for research applications [5]. Essentially, our results suggest a technological boom and associated cost reductions permitted a more diverse group of researchers and engineers with limited expertise to experiment with UAS technology to survey animals for specific applications compared to results before 2015 [7]. In addition to quality imagery, UAS surveys provided substantial advantages over ground or occupied aircraft surveys, which are often more expensive, logistically difficult, or more dangerous for biologists [4, 5, 7]. Consequently, many studies were published in the last 8 years of this fledgling field to validate UAS surveys but used descriptive statistics for typically one species at a time over a limited spatial and temporal scales while not assessing biases. Many of these studies were difficult to find because they were published in lower-impact regional journals, were spread among wildlife biology and engineering journals, or were published in formats less recognized in different fields (i.e., notes or short communications in wildlife biology journals or conference proceedings in engineering journals), all of which may not show up in the publication databases or internet searches that we used.

Despite the advances to UAS technology, our results for animal taxa and environments mirror a literature review performed before 2015 [7]. Most studies in our map still focus on large birds and mammals in open areas, often in aggregations, likely due to the ease and efficiency (e.g., time and cost) of sampling these conditions with UAS compared to traditional survey methods [21, 45, 54–56]. Many biases and constraints were mentioned in studies, and future reviews could focus on quantifying how these factors could influence the counts of animals. One important constraint which influences the use of UAS was UAS regulations, similar to findings in reviews before and at the beginning of the UAS boom in 2015 [5, 7]. Regulations, such as the need to maintain visual line-of-sight or perform flights < 400ft AGL, are necessary to ensure safety given the growing recreational and commercial use of UAS in the National Airspace System. But waivers and authorizations for operation outside these regulations (e.g., https://www.faa.gov/uas/commercial_operators/part_107_waivers), especially for animal research, could be important for future UAS animal studies.

### Implications to inform further research

Our systematic map results also revealed important knowledge gaps in the current literature due to the infancy of UAS technology for animal monitoring. One critical gap is the lack of standardized reporting and best practices for methods using UAS to conduct surveys. Many of the articles in this systematic map failed to report data on methods and only 7 of the 216 articles provided links to data repositories. Barnas et al. [29] established guidelines to report standardized methods in UAS studies to improve transparency and repeatability in UAS studies, an important step for developing best practices. However, studies that address how factors typical in UAS surveys affect bias of animal counts from images, such as differences in UAS platform, sensor, AGL, survey technique, timing, occlusion, landscape, weather and other environmental factors are relatively few or lacking [57–60].

Similarly, another critical gap in the current literature is the use of UAS to survey delineated areas for a community of animal species. Surveying a defined area for an animal community is common practice for many wildlife and conservation applications, such as within state, provincial, or national park boundaries [61, 62], for species of endangered ecosystems [63], after natural or other disasters [64], planning for future human development [65], and to mitigate aircraft-animal collisions with species on and near airports [31, 51, 66]. Despite the importance of community surveys, few studies have investigated best practices for conducting community surveys with a UAS [55, 67, 68].

The literature for using UAS to conduct animal surveys is approaching an important transition point, according to the results of our systematic map. The number of studies published per year have peaked between 40–45 studies for the last three years, but could continue to increase, suggesting that UAS applications will continue as a critical tool for animal surveys. Because most studies have been limited to single-species case studies, future studies should first focus on determining best practices for surveying multiple animal species among diverse conditions with UAS, a fundamental knowledge gap for adopting any new technology [69]. Second, with standardizing best practices for monitoring animals with UAS in mind, defining animal communities of interest and appropriate approaches to sampling them, such as delineating spatial boundaries of interest and appropriate flight patterns for efficiently sampling the area of interest while minimizing sampling error, would further advance the field for common conservation and management applications and make UAS applications comparable to other survey methods [6, 45, 55, 57, 68]. Last, communicating results of successes and failures, as well as biases and constraints, in the development of these UAS applications to avoid repeating pitfalls among research endeavors would further support the advancement of UAS applications and identify situations in which these technologies are best used or best avoided, while leading to less biased, standardized methodologies.

Across geographies, most studies were in open land covers (e.g., grassland, bare, beach, etc.), with few studies in forested systems where animals are often obstructed or occluded by overhead vegetation. Forests deserve research attention, especially for UAS applications, considering, forested systems such as tropical rainforests tend to harbor the greatest terrestrial vertebrate diversity [70, 71] and have urgent conservation [72]. Despite the difficulty of obstructions when surveying forested systems for animals, investigators working in forests could benefit from UAS monitoring approaches, and a few case studies employing UAS with thermal sensors have demonstrated promise for UAS surveys, as well as for nocturnal surveys [57, 73–75].

Thermal sensors and nocturnal surveys have received little attention compared to visible (RGB) sensors. Thermal cameras are often paired or combined with RGB sensors [57, 68, 74]; however, the cost of thermal or dual sensors (RGB + thermal) is typically substantially greater than RGB sensors alone. Further, contrast and resolution are often highlighted as constraints, so determining best practices, limitations, and tradeoffs of thermal or both thermal and RGB sensors can provide important information to augment and complement surveys using only RGB sensors [57, 68, 74] and should be another focus of future studies. A few recent studies also demonstrated that the use of audio sensors to surveys wildlife may be an exciting new avenue of research [46, 47].

Promising avenues of future research include potential software and hardware advances surrounding the methodology for surveying animals with UAS. Both avenues tend to be outside the expertise of most wildlife researchers and necessitate collaborations with engineering professionals [14, 51]. Automated counting of animals from UAS images using machine-learning software holds perhaps the best return on investment to improve animal surveys with UAS, as automated detection, identification, and quantification of animals from UAS images can save time (one of the top constraints noted in animal studies), reduce personnel and costs, and streamline survey logistics [6, 14, 51]. Studies in this field have made impressive advances, from semi- to fully-automated methods, towards the ultimate goal of accurate animal surveys in real-time [6, 14, 76].

Similarly, hardware advances could also improve UAS surveys. For example, time and batteries were listed as two UAS-related constraints for animal surveys in our systematic map and a 2015 review [7]. Currently, batteries constrain most UAS platforms to approximately ≤ 30–40 min of flight time, limiting the area surveyed, particularly with a lawnmower survey pattern. Optimization of batteries to reduce weight and increase flight time would allow for coverage of larger areas and permit pilots to carry larger payloads, improving survey efficiency. Although the goals of real-time animal counts during surveys and hours-long flight times for UAS may still be on the distant horizon, continued and improved collaborations among wildlife biology- and engineering-related disciplines, as well as policy makers, will likely transition future possibilities into tangible options, readily available for UAS researchers worldwide.

## Supplementary Information

**Additional file 1.** Adherence to the ROSES guidelines.

**Additional file 2.** Call for supplementary literature.

**Additional file 3.** List of benchmark articles.

**Additional file 4.** Metadata file for the database.

**Additional file 5.** Search results with decisions at title and abstract level after duplicate removal.

**Additional file 6.** Access database of full text reviewed articles and extracted data for included articles.

**Additional file 7.** Runtime database of full text reviewed articles and extracted data for included articles.

**Additional file 8.** Extracted data for included articles in spreadsheet format.

**Additional file 9.** Spreadsheet of consistency check among reviewers used for calculating Cohen’s kappa coefficients for agreement.

**Additional file 10.** Standard operating procedure for screening articles and extracting data into the Access database.

**Additional file 11.** Frequency of statistical analyses.

**Additional file 12.** Frequency and definitions for biases and constraints.

## Data Availability

All data generated or analyzed during this study are included in this published systematic map or its additional files.
