# Supplementary material for: Evidence on the efficacy of small unoccupied aircraft systems (UAS) as a survey tool for North American terrestrial, vertebrate animals: a systematic map
Source: Environ Evid. 2023 Feb 13;12:3. doi: 10.1186/s13750-022-00294-8 (PMC11378819; doi:10.1186/s13750-022-00294-8)
Supplement: Supplementary file 2 — Additional file 2. Call for supplementary literature. [file 13750_2022_294_MOESM2_ESM.docx]

Read Me

Call for supplementary literature

May 2021

Elmore et al.

To whom it may concern,

We are conducting a literature search for both non and peer reviewed publications to better understand the use of small Unmanned Aircraft Systems to monitor terrestrial wildlife. This effort will be summarized and published as a systematic map ([Environmental Evidence | Systematic map (biomedcentral.com)](https://environmentalevidencejournal.biomedcentral.com/submission-guidelines/preparing-your-manuscript/systematic-map)). If you have conducted such sUAS studies that are unavailable in Web of Science, Scopus, Ebsco, Proquest, or Google Scholar, we would much appreciate you forwarding your research to Mike Curran ([mfc143@msstate.edu](mailto:mfc143@msstate.edu)) or Jared Elmore ([jae133@msstate.edu](mailto:jae133@msstate.edu)).

Examples of literature are peer reviewed publications, government reports, white papers, gray literature, and information from conference proceedings. We have already conducted a thorough online search, so please only forward your research if it is not found in one of the above-mentioned databases.

Thank you in advance.
