# Supplementary material for: Evidence on the efficacy of small unoccupied aircraft systems (UAS) as a survey tool for North American terrestrial, vertebrate animals: a systematic map
Source: Environ Evid. 2023 Feb 13;12:3. doi: 10.1186/s13750-022-00294-8 (PMC11378819; doi:10.1186/s13750-022-00294-8)
Supplement: Supplementary file 3 — Additional file 3. List of benchmark articles. [file 13750_2022_294_MOESM3_ESM.docx]

Read Me

Benchmark Articles

May 2021

Elmore et al.

List of 41 articles on using small Unoccupied Aircraft Vehicles as a survey tool for wildlife from two review articles; review articles are citations 19 and 36.

1. Barasona JA, Mulero-Pázmány M, Acevedo P, Negro JJ, Torres MJ, Gortázar C, Vicente J (2014) Unmanned aircraft systems for studying spatial abundance of ungulates: relevance to spatial epidemiology. PLoS One 9:e115608

2. Chabot D, Bird DM (2015) Wildlife research and management methods in the 21st century: Where do unmanned aircraft fit in? J Unmanned Veh Syst 3:137–155

3. Chrétien L, Théau J, Ménard P (2016) Visible and thermal infrared remote sensing for the detection of white‐tailed deer using an unmanned aerial system. Wildl Soc Bull 40:181–191

4. Christiansen P, Steen KA, Jørgensen RN, Karstoft H (2014) Automated detection and recognition of wildlife using thermal cameras. Sensors 14:13778–13793

5. Christie KS, Gilbert SL, Brown CL, Hatfield M, Hanson L (2016) Unmanned aircraft systems in wildlife research: Current and future applications of a transformative technology. Front Ecol Environ 14:241–251

6. Cliff OM, Fitch R, Sukkarieh S, Saunders DL, Heinsohn R (2015) Online localization of radio-tagged wildlife with an autonomous aerial robot system. Robot Sci Syst

7. Ditmer MA, Vincent JB, Werden LK, Tanner JC, Laske TG, Iaizzo PA, Garshelis DL, Fieberg JR (2015) Bears show a physiological but limited behavioral response to unmanned aerial vehicles. Curr Biol 25:2278–2283

8. Dulava S, Bean WT, Richmond OMW (2015) Environmental reviews and case studies: applications of unmanned aircraft systems (UAS) for waterbird surveys. Environ Pract 17:201–210

9. Elsey RM, Trosclair PL (2016) The use of an unmanned aerial vehicle to locate alligator nests. Southeast Nat 15:76–82

10. Evans IJ, Jones TH, Pang K, Evans MN, Saimin S, Goossens B (2015) Use of drone technology as a tool for behavioral research: a case study of crocodilian nesting. Herpetol Conserv Biol 10:90–98

11. Goebel ME, Perryman WL, Hinke JT, Krause DJ, Hann NA, Gardner S, LeRoi DJ (2015) A small unmanned aerial system for estimating abundance and size of Antarctic predators. Polar Biol 38:619–630

12. Gonzalez LF, Montes GA, Puig E, Johnson S, Mengersen K, Gaston KJ (2016) Unmanned aerial vehicles (UAVs) and artificial intelligence revolutionizing wildlife monitoring and conservation. Sensors 16:97

13. Goodenough AE, Carpenter WS, MacTavish L, MacTavish D, Theron C, Hart AG (2018) Empirically testing the effectiveness of thermal imaging as a tool for identification of large mammals in the African bushveldt. Afr J Ecol 56:51–62

14. Hahn N, Mwakatobe A, Konuche J, de Souza N, Keyyu J, Goss M, Chang’a A, Palminteri S, Dinerstein E, Olson D (2017) Unmanned aerial vehicles mitigate human–elephant conflict on the borders of Tanzanian Parks: a case study. Oryx 51:513–516

15. Han Y-G, Yoo SH, Kwon O (2017) Possibility of applying unmanned aerial vehicle (UAV) and mapping software for the monitoring of waterbirds and their habitats. J Ecol Environ 41:1–7

16. Hodgson JC, Baylis SM, Mott R, Herrod A, Clarke RH (2016) Precision wildlife monitoring using unmanned aerial vehicles. Sci Rep 6:1–7

17. Israel M (2011) A UAV-based roe deer fawn detection system. Int Arch Photogramm Remote Sens 38:1–5

18. IV GPJ, Pearlstine LG, Percival HF (2006) An assessment of small unmanned aerial vehicles for wildlife research. Wildl Soc Bull 34:750–758

19. Jiménez López J, Mulero-Pázmány M (2019) Drones for conservation in protected areas: present and future. Drones 3:10

20. Junda J, Greene E, Bird DM (2015) Proper flight technique for using a small rotary-winged drone aircraft to safely, quickly, and accurately survey raptor nests. J Unmanned Veh Syst 3:222–236

21. Koh LP, Wich SA (2012) Dawn of drone ecology: low-cost autonomous aerial vehicles for conservation. Trop Conserv Sci 5:121–132

22. Linchant J, Lisein J, Semeki J, Lejeune P, Vermeulen C (2015) Are unmanned aircraft systems (UASs) the future of wildlife monitoring? A review of accomplishments and challenges. Mamm Rev 45:239–252

23. Lubow BC, Ransom JI (2016) Practical bias correction in aerial surveys of large mammals: Validation of hybrid double-observer with sightability method against known abundance of feral horse (Equus caballus) populations. PLoS One 11:e0154902

24. McEvoy JF, Hall GP, McDonald PG (2016) Evaluation of unmanned aerial vehicle shape, flight path and camera type for waterfowl surveys: disturbance effects and species recognition. PeerJ 4:e1831

25. Mulero‐Pázmány M, Barasona JÁ, Acevedo P, Vicente J, Negro JJ (2015) Unmanned aircraft systems complement biologging in spatial ecology studies. Ecol Evol 5:4808–4818

26. Mulero-Pázmány M, Jenni-Eiermann S, Strebel N, Sattler T, Negro JJ, Tablado Z (2017) Unmanned aircraft systems as a new source of disturbance for wildlife: A systematic review. PLoS One 12:e0178448

27. Olivares-Mendez MA, Fu C, Ludivig P, Bissyandé TF, Kannan S, Zurad M, Annaiyan A, Voos H, Campoy P (2015) Towards an autonomous vision-based unmanned aerial system against wildlife poachers. Sensors 15:31362–31391

28. Rees AlF, Avens L, Ballorain K, Bevan E, Broderick AC, Carthy RR, Christianen MJA, Duclos G, Heithaus MR, Johnston DW (2018) The potential of unmanned aerial systems for sea turtle research and conservation: a review and future directions. Endanger Species Res 35:81–100

29. Sardà‐Palomera F, Bota G, Padilla N, Brotons L, Sardà F (2017) Unmanned aircraft systems to unravel spatial and temporal factors affecting dynamics of colony formation and nesting success in birds. J Avian Biol 48:1273–1280

30. SARDÀ‐PALOMERA F, Bota G, Viñolo C, Pallarés O, Sazatornil V, Brotons L, Gomáriz S, Sardà F (2012) Fine‐scale bird monitoring from light unmanned aircraft systems. Ibis (Lond 1859) 154:177–183

31. Stark DJ, Vaughan IP, Evans LJ, Kler H, Goossens B (2018) Combining drones and satellite tracking as an effective tool for informing policy change in riparian habitats: a proboscis monkey case study. Remote Sens Ecol Conserv 4:44–52

32. Torney CJ, Dobson AP, Borner F, Lloyd-Jones DJ, Moyer D, Maliti HT, Mwita M, Fredrick H, Borner M, Hopcraft JGC (2016) Assessing rotation-invariant feature classification for automated wildebeest population counts. PLoS One 11:e0156342

33. Tremblay JA, Desrochers A, Aubry Y, Pace P, Bird DM (2017) A low-cost technique for radio-tracking wildlife using a small standard unmanned aerial vehicle. J Unmanned Veh Syst 5:102–108

34. Vas E, Lescroël A, Duriez O, Boguszewski G, Grémillet D (2015) Approaching birds with drones: first experiments and ethical guidelines. Biol Lett 11:20140754

35. Vermeulen C, Lejeune P, Lisein J, Sawadogo P, Bouché P (2013) Unmanned aerial survey of elephants. PLoS One 8:e54700

36. Wang D, Shao Q, Yue H (2019) Surveying wild animals from satellites, manned aircraft and unmanned aerial systems (UASs): A review. Remote Sens 11:1308

37. Webber D, Hui N, Kastner R, Schurgers C (2017) Radio receiver design for unmanned aerial wildlife tracking. In: 2017 Int Conf Comput Netw Commun. IEEE, pp 942–946

38. Weissensteiner MH, Poelstra JW, Wolf JBW (2015) Low‐budget ready‐to‐fly unmanned aerial vehicles: An effective tool for evaluating the nesting status of canopy‐breeding bird species. J Avian Biol 46:425–430

39. Wich S, Dellatore D, Houghton M, Ardi R, Koh LP (2016) A preliminary assessment of using conservation drones for Sumatran orang-utan ( *Pongo abelii* ) distribution and density . J Unmanned Veh Syst 4:45–52

40. Wilson AM, Barr J, Zagorski M (2017) The feasibility of counting songbirds using unmanned aerial vehicles. Auk Ornithol Adv 134:350–362

41. Witczuk J, Pagacz S, Zmarz A, Cypel M (2018) Exploring the feasibility of unmanned aerial vehicles and thermal imaging for ungulate surveys in forests-preliminary results. Int J Remote Sens 39:5504–5521
