# Supplementary material for: Evidence on the efficacy of small unoccupied aircraft systems (UAS) as a survey tool for North American terrestrial, vertebrate animals: a systematic map
Source: Environ Evid. 2023 Feb 13;12:3. doi: 10.1186/s13750-022-00294-8 (PMC11378819; doi:10.1186/s13750-022-00294-8)
Supplement: Supplementary file 4 — Additional file 4. Metadata file for the database. [file 13750_2022_294_MOESM4_ESM.docx]

Read Me

Metadata

September 2022

Elmore et al.

Systematic Map Metadata for all categories of data extraction from articles. This document is specifically relates to Additional file 6, 7, and 8. Colors of text correspond to the different data tables/categories of data in the access database.

- - - 1. Master Table:
         1. Key (Short Text)- Rayyan key
         2. Reviewer- the assigned reviewer for each article
         3. Downloaded (Yes/No)- Whether the article has been downloaded or obtained from library
         4. English (Yes/No)- is the article in English?
         5. Decision (Short Text)- included in systematic map review

Include;Exclude;Maybe

- - - - 1. Author_Names (Long Text)- article authors
        2. Article_Title (Long Text)- title of article
        3. Year_Published (Number)- year of publication
        4. Month_Published (Number)- month of publication
        5. Day_Published (Number)- day of publication
        6. Publication_Type (Short Text)- type of publication

Journal Article;Conference Proceeding;Report;Thesis/Dissertation;Book;Other

- - - - 1. Journal (Short Text)- journal article is published in
        2. ISSN (Short Text)
        3. Volume (Number)- journal volume
        4. Issue (Number)- journal issue
        5. Pages (Short Text)- page range
        6. Peer_Reviewed (Yes/No)- was article peer reviewed?
        7. URL (Long Text)- article url
        8. Complete (Yes/No)- is data entry complete for this article?
        9. Constraints (Long Text)- list any constraints to using sUAS for monitoring animals
        10. Remarks (Long Text)- any additional remarks or info
        11. Potential Duplicate? (Yes/No)- is this article similar to any other article already documented?
      1. Overall_Methods:
         1. Key (Short Text)- Rayyan key
         2. Years_Study (Short Text)- years that the study took place

0 indicates unknown

- - - - 1. Country_of_Study (Short Text)- country where the study took place

Look-up Table: Countries

Note: Antarctica is listed as a country for ease of data collection

- - - - 1. StateOrProvince_of_Study (Short Text)- state/providence where the study took place

Look-up Table: StatesAndProvinces

- - - - 1. General Latitude (Number)- general latitude for study area if provided in article

0 indicates unknown

- - - - 1. General Longitude (Number)- general longitude for study area if provided in article

0 indicates unknown

- - - 1. Statistics:
         1. Stats_ID (Auto Number) - hidden on form
         2. Key (Short Text)- Rayyan key
         3. Bias_Estimation_Method (Short Text)- method used to estimate bias

Look-up Table: Bias_Estimation_Method

Detection-

Overall accuracy-

Consult team members before adding new method

Search document for “detection” and/or “accuracy” and/or “bias”

- - - - 1. Factors_Affecting_Bias (Long Text)- factors listed that affected bias
        2. Description_Other_Method (Short Text)- brief description on other method compared to UAS flights

Look-Up Table: Description_Other_Method

- - - - 1. Type_Of_Analysis (Short Text)- brief description of analysis

Look-up Table: Statistical_Analyses

Only statistics that are looking at counting and/or comparison to other methods and/or bias and/or occupancy model development. Be as specific as you want to be

- - - - 1. Raw_Data (Long Text)- Link to data if available for download

If data is not available, enter “NA”

- - - 1. Individual_Methods:
         1. Method_ID (Auto Number) - hidden on form
         2. Key (Short Text)- Rayyan key
         3. Landcover_Type (Short Text)- what type of specific landcover did the study take place in?

Look-up Table: Landcover_Type

- - - - 1. Flight_Time_Of_Day (Short Text)- start time of flights

Look-up Table: Time_of_Day

- - - - 1. Purpose_Of_Study (Short Text)- what was the purpose of the study?

Look-up Table: Purpose_Of_Study

Default set to: Monitoring/Counting

- - - - 1. Ground_Control_Points_Used (Short Text)- were ground control points used?

i.e. Something in the imagery has a known GPS that was recorded

- - - - 1. Ground_Truth (Short Text)- was there on-the-ground verification of what wildlife was at the site?

Yes;No;Unknown

Yes means that they definitely know how many animals were observed with drone with 100% accuracy (i.e. decoys, captive animals)

- - - 1. Drones:
         1. Drone_ID (Auto-number) - hidden on form
         2. Key (Short Text)- Rayyan key
         3. Drone_Manufacturer (Short Text)- manufacturer of drone

Look-up Table: Drone_Manufacturer_No_Duplicates

- - - - 1. Drone_Model (Short Text)- model of drone

Look-up Table: Drones

- - - - 1. Control_Type (Short Text)- how was drone controlled?

Automatic;Manual;Unknown

- - - - 1. Gimbal (Short Text)- was a gimbal used?

Yes;No;Unknown

If article does not mention a gimbal, then enter as “Unknown”

- - - - 1. Flight_Software (Short Text)- type of UAS software used

Look-up Table: Flight_Software

Unknown is when article don’t state if software was used or software type was not listed

NA means they explicitly stated that they did not use software (or that flew manually).

- - - - 1. AGL (Number)- above ground level reported in meters. Above Ground Level

Record individual values if listed

Record median if a range is listed

If listed as less than, record as median and note this in Remarks section

- - - - 1. Flight_Speed (Number)- flight speed reported in m/s

If multiple are reported, record median value

- - - - 1. Flight_Pattern (Short Text)- how did they fly?

Look-up Table: Flight_Pattern

Record parallel transects as “Lawnmower”

- - - - 1. Flight_Duration (Number)- number of minutes of average flight

0 indicates unknown

- - - 1. Sensors:
         1. Sensor_ID (Auto-number) - hidden on form
         2. Key (Short Text)- Rayyan key
         3. Sensor_Manufacturer (Short Text)- manufacturer of sensor

Look-up Table: Sensor_Manufacturer_No_Duplicates

- - - - 1. Sensor_Model (Short Text)- model of sensor

Look-up Table: Sensors

- - - - 1. Field_Calibration (Short Text)- was calibration conducted in the field?

Yes;No;Unknown

- - - - 1. Calibration_Type (Short Text)- how was calibration conducted?

Automated;Manual;Combination;Unknown;Not Applicable

- - - - 1. Image_Analysis (Short Text)- how were images analyzed?

Computer;Human;Combination;Unknown

- - - - 1. Image_Preprocessing (Short Text)- were images processed before analysis?

Yes;No;Unknown

- - - - 1. Sound? (Yes/No)- did sensor use animal noises for monitoring?
      1. Animals:
         1. Animal_ID (Auto-number) - hidden on form
         2. Key (Short Text)- Rayyan key
         3. Scientific_Name (Short Text)- scientific name of animal

Look-up Table: Animals

Derived from: [BirdLife Data Zone](http://datazone.birdlife.org/species/taxonomy) and [Global Mammal Checklist - Mammal Watching](https://www.mammalwatching.com/resources/global-mammal-checklist/)

Consult team members before adding new species

- - - 1. Locations:
         1. Indiv_Location_ID (Auto-number) - hidden on form
         2. Key (Short Text)- Rayyan key
         3. Latitude (Number)- specific latitude
         4. Longitude (Number)- specific longitude
