# Supplementary material for: Evidence on the efficacy of small unoccupied aircraft systems (UAS) as a survey tool for North American terrestrial, vertebrate animals: a systematic map
Source: Environ Evid. 2023 Feb 13;12:3. doi: 10.1186/s13750-022-00294-8 (PMC11378819; doi:10.1186/s13750-022-00294-8)
Supplement: Supplementary file 10 — Additional file 10. Standard operating procedure for screening articles and extracting data into the Access database. [file 13750_2022_294_MOESM10_ESM.docx]

Read Me

Standard operating proceedure

September 2022

Elmore et al.

SOP used by reviewers JAE, LRJ, and EAS for systematic map articles post title and abstract screening.

1. Downloading full text:
   1. In the MSU_UAV_Phase1 shared folder find the Full_text_articles_systmap subfolder. There you can open the Rayyan_exported_articles.xlsx sheet
   2. Look for articles that have not been downloaded
   3. Download a pdf copy of each and mark “Yes” when it is downloaded
      1. If you cannot find it available, either request from the library or corresponding author and mark requested
   4. Once downloaded, save using naming convention of author_year to the Downloaded_articles subfolder.
2. Identify your assigned records and keep track of tasks
   1. Open Rayyan_exported_articles.xlsx sheet
   2. Locate your name as reviewer
   3. Use this color scheme for identifying task completion:
      1. Pdf download not found- search more or re-download
      2. Pdf saved in Mendeley and citation information checked and/or corrected
      3. Read article, highlighted important data, and added tags in Rayyan
      4. Data fully entered into Access database
      5. Found, but in foreign language
3. Mendley Tasks:
   - 1. Open Mendeley and highlight/open/click on the group project (UAS systematic map)
     2. Add article to Mendeley group project using Add feature in top left-hand corner
     3. Fix any citation details that are incorrect
     4. Keep track of status of article review
        1. Green Dot- not read
        2. Grey Dot- read, annotated (highlighted), citation checked, tags noted, and entered into Rayyan
        3. Starred- fully entered into Access database
     5. Include the following in the Tags section of Mendley:
        1. Rayyan key (“rayyan-000000000”)
        2. “Include” or “Exclude”
        3. New Rayyan tags:

| Machine learning | Computer vision | Bias | Behavior | Review |
| --- | --- | --- | --- | --- |

1. Article Review
   1. Read full text by opening .pdf in Mendley
      1. Be sure to sync Mendley each time before opening and closing so that it will sync with other devices
   2. Make note of:
      - 1. Constraints and remarks mentioned in the article with orange highlighter
        2. Overall_Methods:
           1. Years_Study
           2. Country_of_Study
           3. StateOrProvince_of_Study
           4. General Latitude
           5. General Longitude
        3. Statistics:
           1. Bias_Estimation_Method
           2. Factors_Affecting_Bias
           3. Description_Other_Method
           4. Type_Of_Analysis
           5. Raw_Data
        4. Individual_Methods:
           1. Landcover_Type
           2. Flight_Time_Of_Day
           3. Purpose_Of_Study
           4. Ground_Control_Points_Used
           5. Ground_Truth
        5. Drones:
           1. Drone_Manufacturer
           2. Drone_Model
           3. Control_Type
           4. Gimbal
           5. Flight Software
           6. AGL: Above_Ground_Level
           7. Flight_Speed
           8. Flight_Pattern
           9. Flight_Duration (Note “0” = Unknown)
        6. Sensors:
           1. Sensor_Manufacturer
           2. Sensor_Model
           3. Field_Calibration
           4. Calibration_Type
           5. Image_Analysis
           6. Image_Preprocessing
           7. Sound?
        7. Animals:
           1. Scientific_Name
        8. Behavioral response information
           1. Highlight sections related to behavior
        9. Tags/Labels (for Rayyan)
           1. Type these in the Tags section of Mendeley
      1. Determine inclusion or exclusion
         1. Make note of this in Mendeley tags field (so that you can enter this into Access later)
2. Update Mendeley if more information should be added in Tags section after full text review
3. Update tags in Rayyan:
   1. Open: [Rayyan](https://rayyan.ai/users/sign_in)
   2. Search Rayyan key (number only in top right)
   3. Enter new tags under Label, then select Enter
   4. Ensure that Label was entered for the correct article
4. Access Data Entry:

Important Notes:

- - Refer to Systematic_Map_Metadata Word doc for specifics on what each of these fields means
  - To check on uncheck a box in Access, use the spacebar
  - If drop-down menu does not have item that you would like to add, keep the form open, select the appropriate look-up table under Tables on the left side of screen (refer to Metadata document for look-up table names), open look-up table, and add new record item. Save and close look-up table. Type field name in just as you entered on look-up table (note that it will not auto-populate, but you will be able to save)
  - NOT DO USE commas in text fields
  - Be sure to enter “NA” if no data for a particular field- no field should be left blank
  - If article does not describe specifically what we are inquiring about, then list as Unknown
  1. Reserve a time to use the Access database on the Systematic Map Access Database Shared Calendar
     1. Please remember to close the access database wherever you are not working on it and do not have it open if you do not have a time reserved on the calendar
  2. Open MSU_USDA_Phase1_Map Access database
  3. Navigation Pane will be displayed
     1. If not displayed, scroll to Forms section on left side of screen and select “Navigation Pane”
  4. Select Edit Master Table Data
     1. Copy the Rayyan key from the Mendeley tag field for the article you will be entering data for
     2. Paste this Rayyan key into the “Find Key” box
     3. Press Enter
        1. Data should then pull up that corresponds to that article
     4. Press tab to move through the fields and be sure to complete:
        1. Publication Type
        2. Peer_Reviewed
        3. Potential Duplicate for any articles very similar to one another
     5. Be sure to update the “Decision” field from your notes that you recorded in your Mendeley tag section for this article
        1. If decision is changed to “exclude”, add the reason for exclusion to the “Remarks” field
        2. If excluded, no other data needs to be entered in access for this article
     6. Select “Save”
     7. Select “Close Form”
  5. Press Tab then Enter to move to Enter Overall Methods Data
     1. Select Add New Record
     2. Fill in information
        1. If separate years of study, enter as separate overall methods
     3. Note latitude and longitude if provided in article
        1. If separate study sites, enter as separate overall methods
        2. If no latitude and longitude provided in text, do not include
     4. Select “Save”
     5. Select “Close Form”
  6. Press Tab then Enter to move to Enter Statistics Data
     1. Select Add New Record
     2. Fill in information
     3. Select “Save”
     4. Select “Add Another Statistical Analysis” if you have another analysis to enter
     5. Select “Close Form”
  7. Press Tab then Enter to move to Enter Individual Methods
     1. Select Add New Record
     2. Fill in information
     3. Select “Save”
     4. Select “Add Another Method” if you have another analysis to enter
     5. Select “Close Form”
  8. Press Tab then Enter to move to Enter Drone Data
     1. Select Add New Record
     2. Fill in information
     3. If you need to enter another drone or Custom Drone:
        1. Be sure to add to both to the “Drones” and “Dones_Manufacturer_No_Duplicates” look-up tables
        2. Manufacturer for Custom Drone = “Custom”
        3. Drone Model for Custom Drone = Last name of first author of article (et al. if necessary) followed by publication year
     4. All AGLs should be entered as separate records if altitudes were treatment levels
     5. If a range is listed for flight speed or flight duration, record the median value. 0 means “0” (for point count) or unreported
     6. Select “Save”
     7. Select “Add Another Drone” if you have another analysis to enter
     8. Select “Close Form”
  9. Press Tab then Enter to move to Enter Sensor Data
     1. Select Add New Record
     2. Fill in information
     3. Image Analysis: Combination means both humans and computers analyzed imagery
     4. If you need to enter another sensor:
        1. Be sure to add to both to the “Sensors” and “Sensors_Manufacturer_No_Duplicates” look-up tables
     5. Select “Save”
     6. Select “Add Another Sensor” if you have another analysis to enter
     7. Select “Close Form”
  10. Press Tab then Enter to move to Enter Animal Data
      1. Select Add New Record
      2. Fill in information
      3. Select “Save”
      4. Select “Add Another Animal” if you have another analysis to enter
      5. Select “Close Form”
  11. Press Tab then Enter to move to Relationship Joining
      1. Paste Rayyan Key in “Key” field
      2. Press Enter
         1. Data should then display in each of the sub-tables that corresponds to information that particular article (except subRelationshipJoining)
      3. In the table on the bottom right (subRelationshipJoining), scoll to the bottom of the table (blank row) with the star that indicates New Record
      4. Paste the Rayyan Key into the new field
      5. Copy or type the ID fields from each of the tables into the below corresponding columns matching up to the methods that were described in the article
      6. In the top left (subMaster_Table) check the Complete box and make sure that Decision is correctly assigned
      7. Select “Save”
      8. Select “Close Form”
  12. Data entry for that record is now complete

1. Queries
   1. Be sure to run qryAllDataNoIDAutoNumbers before running any other queries based off of the table that this query creates
