# Supplementary figures and images for: Evidence on the efficacy of small unoccupied aircraft systems (UAS) as a survey tool for North American terrestrial, vertebrate animals: a systematic map
Source: Environ Evid. 2023 Feb 13;12:3. doi: 10.1186/s13750-022-00294-8 (PMC11378819; doi:10.1186/s13750-022-00294-8)

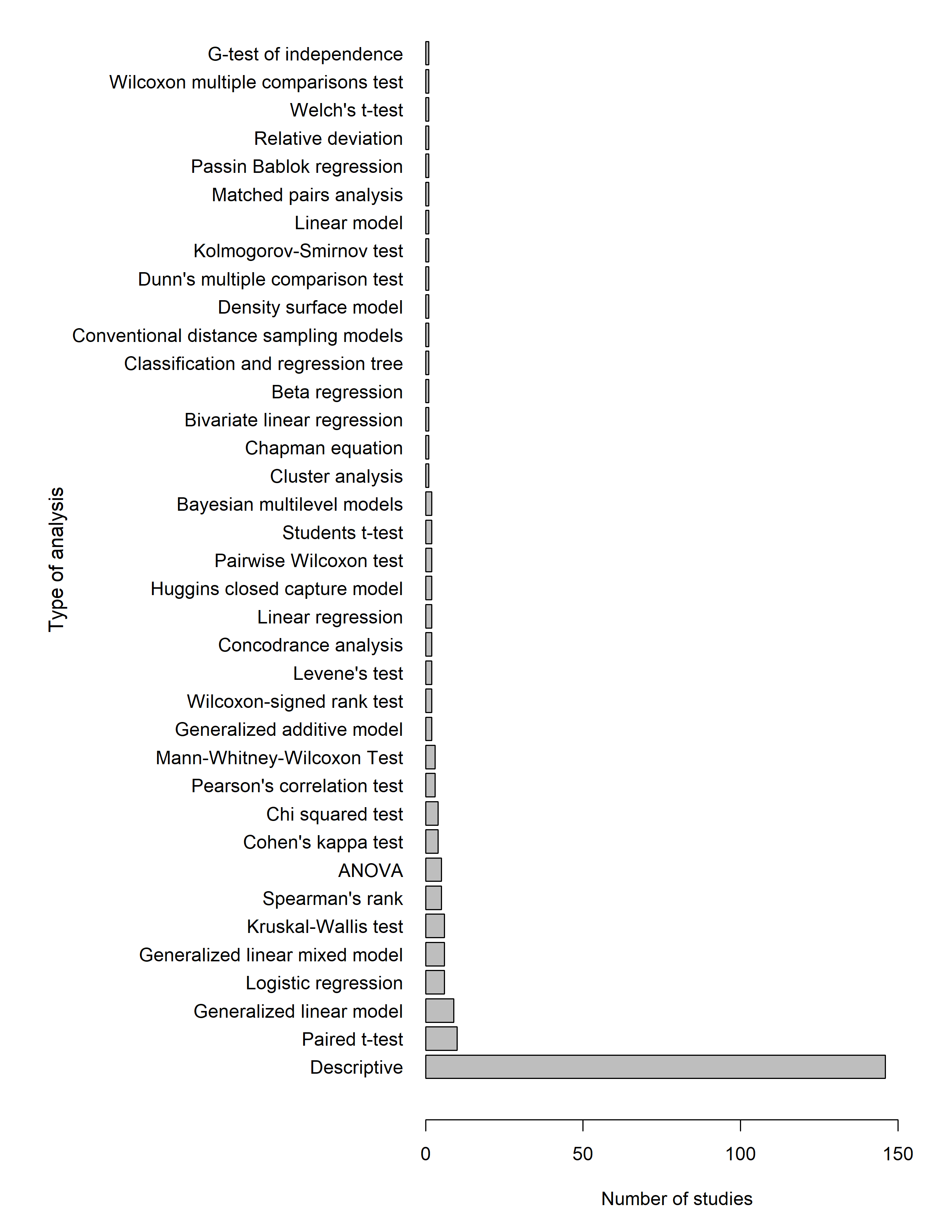

Supplement: Supplementary file 11 — Additional file 11. Frequency of statistical analyses. [file 13750_2022_294_MOESM11_ESM.tiff]
